# Case report: *Coxiella burnetii* endocarditis in the absence of evident exposure

**DOI:** 10.3389/fmed.2023.1220205

**Published:** 2023-08-04

**Authors:** Silvia Bozza, Alessandro Graziani, Monica Borghi, Daniele Marini, Michele Duranti, Barbara Camilloni

**Affiliations:** ^1^Microbiology and Clinical Microbiology Section, Department of Medicine and Surgery, University of Perugia, Perugia, Italy; ^2^Microbiology Unit, Santa Maria della Misericordia Hospital, Perugia, Italy; ^3^Istituto Zooprofilattico Sperimentale dell’Umbria e delle Marche, Perugia, Italy; ^4^Department of Diagnostic Imaging, Santa Maria della Misericordia Hospital, Perugia, Italy

**Keywords:** *Coxiella burnetii*, endocarditis, negative blood culture, Q fever, serological diagnosis

## Abstract

Q fever is a worldwide zoonotic disease caused by *Coxiella burnetii*. In humans, it can manifest clinically as an acute or chronic disease and endocarditis, the most frequent complication of chronic Q fever is associated with the greatest morbidity and mortality. We report a severe case of endocarditis in a 55-year-old man with a history of aortic valve replacement affected by monoclonal gammopathy of undetermined significance (MGUS), and living in a non-endemic area for *C. burnetii*. After two episodes of fever of unknown origin (FUO), occurring 2 years apart and characterized by negative blood cultures, a serological diagnosis of Q fever endocarditis was performed even though the patient did not refer to possible past exposure to *C. burnetii*. Since people with preexisting valvular heart disease, when infected with *C. burnetii*, have reported a 40% risk of Q fever endocarditis, clinicians should maintain a high index of suspicion for infective endocarditis in all patients with FUO even when the exposure to *C. burnetii* appears to be unlikely.

## Introduction

*Coxiella burnetii* is an intracellular Gram-negative bacterium cause of the zoonotic infection known as Q fever. Because Q fever is rarely a notifiable disease, the incidence of human Q fever cannot be assessed in most countries. Current epidemiological studies indicate, however, that Q fever should be considered a public health problem in many countries, including France, the United Kingdom, Italy, Spain, Germany, Israel, Greece, and Canada (Nova Scotia), as well as in many countries where Q fever is prevalent but unrecognized because of poor surveillance of the disease (1). Domestic ruminants are the main human reservoir for this bacterium (1), and infection in humans usually occurs by inhalation of bacteria-laden dust from animal-infected biological materials such as urine, feces, placenta, and amniotic liquid. Individuals in a professional occupation with domestic ruminants, such as veterinarians and livestock farmers, are more frequently exposed than the general population. For this reason, Q fever is predominantly deemed an occupational disease (2, 3).

Ingestion of raw milk and milk products is another alternative route of transmission, while person-to-person transmission is rare (4, 5).

Q fever has two different clinical manifestations: acute and chronic. The first one is mostly characterized by asymptomatic infection or self-limiting influenza-like syndrome (6). Chronic Q fever is the most rare and serious form of the disease, which might occur even after years or decades from the initial acute infection. Certain conditions like preexisting cardiac valve defects and aortic aneurysms are considered risk factors for Q fever and could predispose individuals to the chronic form and its main clinical complication known as infective endocarditis (78%) (4, 7, 8).

During the infection, *C. burnetii* undergoes an antigenic switch causing two different antibodies phase production that helps to differentiate between the acute and chronic forms. During acute infection, phase II antibodies are predominant while a chronic infection is characterized by a rising titer of phase I IgG antibodies. Thus, diagnosis in humans is most commonly made using a serodiagnostic technique like ImmunoFluorescence Assays (IFA) (9).

Here, we report a case of endocarditis due to *C. burnetii* infection diagnosed after two episodes of fever of unknown origin (FUO), occurring 2 years apart, in a patient with a history of cardiac valve replacement and Monoclonal Gammopathy of Undetermined Significance (MGUS).

## Case description

A 55-year-old male patient was admitted in 2019 to Foligno Hospital (Umbria, Italy) for FUO. The patient had a clinical history of an aortic valve replacement (2009), myxoid liposarcoma exeresis (2011), and a diagnosis of MGUS in 2016. Aortic valve replacement was performed for ascending aortic regurgitation and aneurysm. No family history of valve dysfunction was reported. The intermittent fever started 1 month before and was accompanied by shaking chills, night sweats, splenomegaly, and myalgias. At the time of admission, the patient referred to an analogous episode of FUO characterized by the same symptoms in 2017, and the medical team had not found a certain diagnosis. The patient was treated empirically, the symptoms regressed, and then he was discharged from the hospital.

In 2019, the new episode of FUO was associated with negatives blood and urine cultures, but considering the global patient’s clinical picture, the medical staff started a new empiric therapy based on teicoplanin (600 mg per day) and meropenem (1 gr/tid). The patient clinically deteriorated and was transferred to Perugia Hospital (Umbria, Italy).

Once in Perugia Hospital, laboratory results showed anemia, moderate cytopenia, elevated erythrocytes sedimentation rate (ESR), polyclonal hypergammaglobulinemia and thrombocytopenia. C-reactive protein (CRP) and procalcitonin (PCT) were moderately high. Moreover, autoantibodies, Rheumatoid factor (RF) test, and complement components C3 and C4 were measured. As reported in Table 1, Antinuclear Autoantibodies (ANA) titer was 1:160, RF level was 219.1 UI/ml, and C3 was slightly reduced (73 mg/dL). Because it has been described in the literature that high Abs titers toward ANAs have been frequently detected in nonautoimmune individuals with bacterial or viral infection (10), an infective cause was considered.

**Table 1:** Laboratory results upon admission at Perugia Hospital.

Laboratory measurement	Result	Normal range	Unit of measurement
White blood cells	2.89*	3.60–9.60	×10^3^
Red blood cells	3.99*	4.30–5.80	×10^6^
Hemoglobin	10.0*	13.0–17.0	g/dL
Hematocrit	30.4*	38.0–52.0	%
MCV	76.2*	82.0–97.0	fL
MCH	25.1*	27.0–33.0	pg
MCHC	32.9	32.0–36.0	g/dL
RDW	15.6*	11.6–14.5	%
Platelets	112*	140–440	×1,000/UL
MPV	11.1	8.0–13.0	fL
Lymphocytes	29.4	20.5–51.5	%
Monocyte	7.3	1.0–10.0	%
Neutrophils	62.7	42.0–75.0	%
Eosinophils	0.0	0.0–5.0	%
Basophils	0.2	0.0–1.0	%
PT	14.4*	9.8–14.3	sec
INR	1.24*	0.80–1.20	sec
PTT Ratio	1.21*	0.80–1.20	sec
ESR	64*	1–25	1^ h
CRP	1.4*	0–0.5	mg/dL
		Systemic infection: <0.5 improbable	
PCT	1.52	0.5–2.0 possible	ng/mL
		2.0–10 probable	
		>10 septic shock	
ANA	1:160*	Negative	
Anti-nDNA Ab	<1:10	Negative	
C3	73*	90–180	mg/dL
C4	39	10–40	mg/dL
Rheumatoid factor test	219.1*	0.0–14.0	UI/mL
Albumin	46.0*	55.8–66.1	%
Alfa1	6.8*	2.9–4.9	%
Alfa2	7.3	7.1–11.8	%
Beta1	5.4	4.7–7.2	%
Beta2	5.1	3.2–6.5	%
Gamma	29.4*	11.8–18.8	%

*Values out of normal range.

Blood and urine cultures were again tested but still came back negative. Several different pathogens were investigated through serological analysis. Tests for *Legionella pneumophila*, *Treponema pallidum*, Coxsackie virus A and B, Parvovirus B19, *Leishmania* spp., *Toxoplasma gondii*, HIV, and Hepatitis B and C were negative. Epstein-Barr Virus (EBV) serology revealed a past infection although peripheral blood molecular testing indicated potential EBV reactivation as 3,359 DNA copies/mL were detected.

Suspecting an infection of the aortic valve prostheses, transthoracic echocardiogram, transesophageal ultrasound, and chest Computed Tomography (CT) with contrast medium and Positron Emission Tomography-Computed Tomography (PET-CT) were performed. The transthoracic echocardiogram did not show vegetation or valvular dysfunction.

The transesophageal cardiography detected the presence of numerous floating prosthetic strands in the left ventricular outflow tract. No evidence of vegetation was found even if the evaluation was limited by a prosthesis shadow cone. Redundant, thickened, mildly prolapsed mitral flaps with mild regurgitation were detected. Evidence of valve vegetation or pericardial effusion was not detected. The CT showed outcomes of surgery for mechanical prosthesis of ascending aorta.

**Figure 1:**
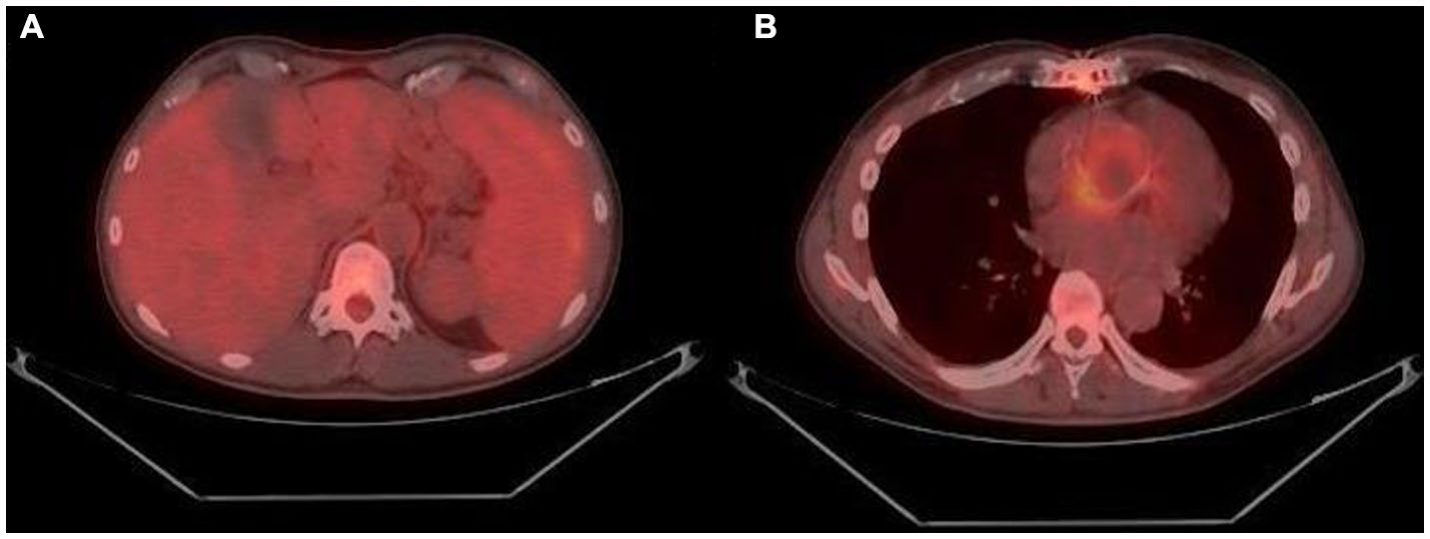
The results of PET-CT: splenic medulla **(A)** and ascending aorta (B).

The PET-CT showed a picture compatible with periaortitis. An intensive uptake of the splenic medulla (Figure 1A) and a periprosthetic uptake at the level of the ascending aorta (Figure 1B) were found, compatible with a reactive-infectious condition and/or hematological disease.

Considering the patient's clinical history, neither of the two hypotheses could be excluded and for this reason, a bone marrow biopsy (BMB) was performed.

The histological report of the BMB confirmed a pathological picture associated with the previous diagnosis of MGUS. Moreover, BMB highlighted the presence of epithelioid microgranulomas compatible with formations typically found in a *C. burnetii* infection. This hypothesis could be supported by the autoimmunity laboratory results because high levels of RF and ANA are frequently found in chronic Q fever (11).

Considering plausible infective endocarditis from non-culturable germs, an immunofluorescence assay (Fuller Laboratories, California) targeting *C. burnetii* phase I and II antibodies was carried out. High titers of Immunoglobulin G and M, both against anti-phase I and II of *C. burnetii* were detected (phase I IgM 3,200, phase II IgM 800, phase I IgG 3,200, and phase II IgG 3,200).

Based on one major radiological criterion (specific uptake of the tracer on the vascular prosthesis in PT-CT) and three minor ones (body temperature > 38.5°C, serology for *C. burnetii* with phase I IgG ≥800 and < 6,400, and predisposing vascular conditions) (12), the suspicion of chronic Q fever was confirmed. For this reason, a targeted antibiotic therapy including doxycycline (100 mg/bid) and hydroxychloroquine (200 mg/tid) was started for at least 24 months (13). The patient was discharged with instructions to carry out scheduled check-ups.

**Figure 2:**
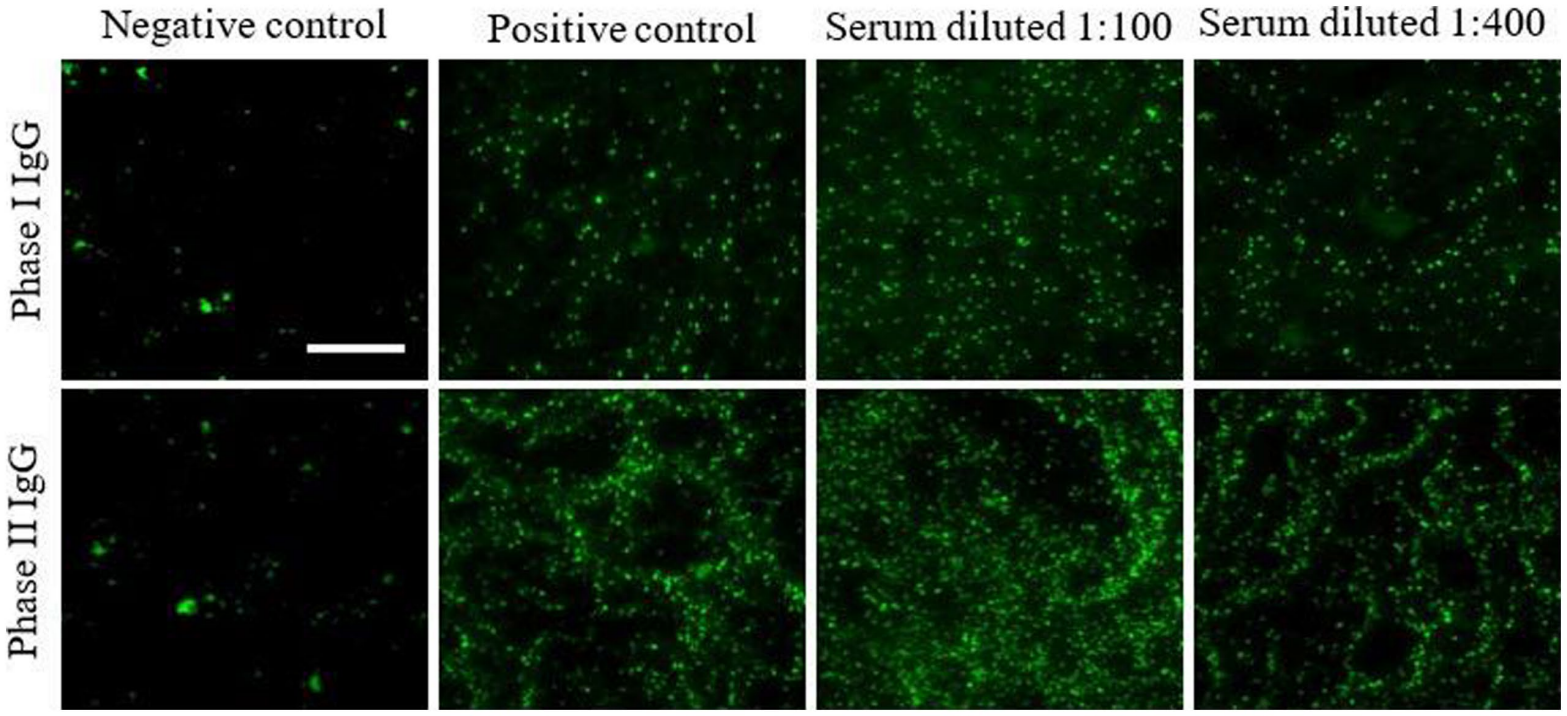
Persistent high titers of phases I and II IgG in patient’s serum after 3-year follow-up. Phases I and II antibodies were determined by ImmunoFluorescence Assays (IFA) on the patient’s serum tested at different dilutions. Images were acquired using a fluorescence high-resolution Microscopy Olympus DP71 with a 40× objective and the analySIS image processing software.

With a 3-year follow-up, the serological tests remained positive for phase I and phase II IgG while the phase II IgM titer showed a decreasing trend up to a negative result. In particular, phase I and II IgG titers showed an oscillatory trend over time probably due to the effects of long-term antibiotic therapy (Figure 2 and Table 2) (14).

**Table 2:** Results of IFA tests during 3-year follow-up.

Antibody titer (IFA)											
		20 days*	2 mo	5 mo	12 mo	16 mo	20 mo	24 mo	29 mo	32 mo	36 mo
Phase I	IgM	3,200	1,600	1,600	1,600	3,200	200	400	400	200	400
	IgG	3,200	25,600	6,400	3,200	1,600	3,200	3,200	3,200	3,200	6,400
Phase II	IgM	800	200	100	100	200	50	<50	<50	<50	<50
	IgG	3,200	25,600	25,600	12,800	12,800	12,800	12,800	6,400	6,400	12,800

*Days/months after hospitalization.

## Discussion

Based on the literature, *C. burnetii* infections are primarily encountered as single cases or discrete clusters of infections associated with a known community outbreak (15). According to the last European Food Safety Authority (EFSA) report, in Europe, 460 cases of human Q fever were confirmed in 2021; no case was reported in Italy, where the last infections (six cases) occurred in 2019 (16). The lack of attention to the diagnosis of *C. burnetii* infection is probably the cause of the underreporting of cases. In our region (Umbria, Central Italy), there are less than one million inhabitants, and is not considered an endemic area for Q fever to date. However, in 2022 two other cases of infective endocarditis from *C. burnetii* were serologically diagnosed, both characterized by negative blood cultures. In accordance with epidemiological studies (12, 17), also our patients are men of over 55 years and with previous heart valve replacement surgery. Interestingly, none of them have carried out or is carrying out jobs typically considered at risk for this infection, and they live in different cities, making it difficult to identify the source of the infection. Despite the low incidence and the predominantly asymptomatic course of the pathology, the possibility of the onset of long-term complications, even as serious as endocarditis, should not be underestimated. For these reasons, since 2015, the European Society of Cardiology guidelines report that a systematic serological test is recommended in all patients with risk factors for chronic Q fever and blood-culture-negative endocarditis (18, 19). Although the clinical picture of the patient at the time of the first episode of FUO with negative blood cultures met the criteria set by the European Society of Cardiology, serological testing for *C. burnetii* was not performed. The febrile episode was treated with non-targeted antibiotic therapy which only temporarily resolved the symptoms but over time may have favored the onset of the chronic form of the pathology, with the development of endocarditis (7). Certainly, as reported in other countries, the low incidence of Q fever in Italy has contributed, also in this case, to the late diagnosis (18).

In conclusion, Q fever could be probably considered an underestimated disease in Italy and so it is important to sensitize doctors about this specific approach to be adopted in patients presenting risk factors and febrile episodes of unknown origin. This approach can anticipate the timing of the diagnosis and promptly adopt a suitable antibiotic therapy to improve the course of the pathology trying to avoid the onset of the chronic form. The presence of Q fever in our rural territory is evidenced by the increasing number of diagnosed cases. For this reason, *C. burnetii* infection should be considered a health issue and, according to OneHealth strategy, a surveillance program should be implemented, both in the human and veterinary context.

## Data availability statement

The datasets presented in this article are not readily available because patient privacy. The data presented in this study are available on request from the corresponding author. Requests to access the datasets should be directed to barbara.camilloni@unipg.it.

## Ethics statement

Ethical review and approval was not required for the study on human participants in accordance with the local legislation and institutional requirements. The patients/participants provided their written informed consent to participate in this study.

## Author contributions

BC and SB conceptualized the study and revised the manuscript. AG, MB, and DM performed the experimental analysis and wrote the original draft. MD performed the radiological investigations. All authors have read and agreed to the published version of the manuscript.

## Conflict of interest

The authors declare that the research was conducted in the absence of any commercial or financial relationships that could be construed as a potential conflict of interest.

## Publisher’s note

All claims expressed in this article are solely those of the authors and do not necessarily represent those of their affiliated organizations, or those of the publisher, the editors and the reviewers. Any product that may be evaluated in this article, or claim that may be made by its manufacturer, is not guaranteed or endorsed by the publisher.
